# Role of polycrystalline F–SnO_2_ substrate topography in formation mechanism and morphology of Pt nanoparticles by solid-state-dewetting†

**DOI:** 10.1039/d5nr00729a

**Published:** 2025-05-15

**Authors:** M. Dierner, S. Peters, M. Wu, C. Rubach, S. Harsha, R. K. Sharma, Z. Y. Siah, M. T. Abudukade, S. Ng, E. Spiecker, M. Altomare, J. Will

**Affiliations:** a Institute of Micro- and Nanostructure Research & Center for Nanoanalysis and Electron Microscopy (CENEM), Friedrich-Alexander-Universität Erlangen-Nürnberg, IZNF Cauerstraße 3 91058 Erlangen Germany martin.dierner@fau.de johannes.will@fau.de sophia.peters@fau.de mingjian.wu@fau.de carmen.rubach@fau.de erdmann.spiecker@fau.de; b Department of Chemical Engineering, MESA+ Institute of Nanotechnology, University of Twente Drienerlolaan 5 7522 NB Enschede The Netherlands s.harsha@utwente.nl r.k.sharma@utwente.nl m.r.abudukade@utwente.nl m.altomare@utwente.nl; c Department of Chemistry and Pharmacy, Friedrich-Alexander-Universität Erlangen-Nürnberg Egerlandstraße 3 91058 Erlangen Germany zayn.siah@fau.de siowwoon.ng@fau.de

## Abstract

Solid-state-dewetting (SSD) of thin films is increasingly utilized to fabricate nanoparticles for catalysis. In-depth understanding of particle formation mechanism is crucial to control key properties of catalytic particles such as size, size distribution, and structure. In contrast to most studies on SSD of thin metal films on smooth substrates (*e.g.*, SiO_2_/Si, …), here we investigate how the topography of practical substrates, such as electrically conductive F-SnO_2_ (FTO), affects the formation mechanism and size of Pt particles – with potential use as nanoparticle electrodes, *e.g.*, in electrochemical conversion or sensing applications. For this, we combined *in situ* scanning transmission electron microscopy (STEM) with *ex situ* rapid thermal annealing (RTA) methodologies. Our results indicate that, by dewetting 5 nm of Pt films on FTO, the arrangement of Pt nanoparticles exhibits a bimodal particle distribution. This is driven by: (i) a thinner initial Pt film thickness in the “depths” of the FTO substrate due to shadowing effects, and (ii) the formation of varying surface curvatures in the Pt film, both caused the topography and grain structure of the FTO substrate. Particularly, the latter introduces an additional driving force for Pt diffusion from peaks and ridges (positive local curvature) to flat terraces (no curvature) and valleys (negative local curvature).

## Abstract 2

SSD of thin films is increasingly utilized to fabricate nanoparticles for catalysis. RTA is combined with plan-view sample preparation for *in situ* TEM to provide insights into the initial stages of the SSD mechanism and formation of Pt NPs on FTO.

## Graphical abstract

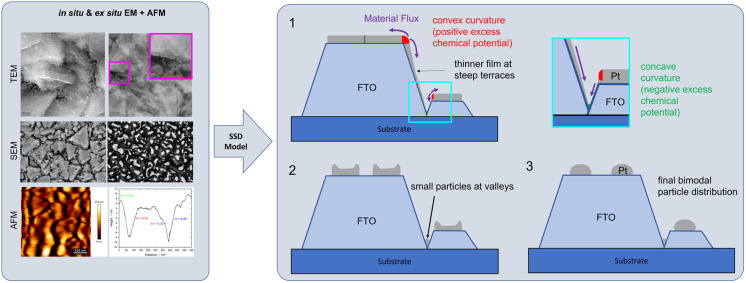

## 1. Introduction

Solid-state-dewetting (SSD) is a process in which a thin film deposited onto a substrate transforms into isolated particles when exposed to temperatures below the film's melting point.^1,2^ Initially considered an undesired phenomenon,^3^ SSD is now recognized as an efficient method for fabricating supported pristine nanoparticles (NPs). This approach has found applications in various fields, including plasmonics,^4,5^ photocatalysis,^6–8^ and more recently, electrocatalysis.^9,10^ In catalysis, a high surface-to-volume ratio for the catalyst material is typically desired, which makes the control over the catalyst particle size and structure crucial. Recently, it has also been shown that the integral length of the contact line between catalyst particles and the substrate plays a significant role in the performance of dewetted NPs, *e.g.*, in electrocatalysis, due to metal–support interaction which can alter the electronic structure of the catalyst, and therefore its catalytic activity.^9^ Therefore, it is essential to precisely control size, structure, and density of the dewetted nanostructures for optimal functionality. In this context, the two most exploited and investigated parameters are the thickness of the as-deposited film^1,9,11^ and the SSD temperature,^12^ where thinner films and higher SSD temperatures lead to smaller NPs. Beyond these factors, the substrate itself can influence the dewetting mechanism,^13^ especially its topography as shown by Wang *et al.*^14^ and Sharipova *et al.*^15^ In the former study, the authors compared particles formed on flat substrates with those formed on pre-patterned substrates featuring inverted pyramidal pit and circular hole patterns. The authors summarized that pre-patterning of the substrate introduces localized (defined) rupture points for the film due to the substrate surface curvature, additional to intrinsic rupture points like grain boundaries in polycrystalline films. This results in a significant reduction in particle size and particle spacing.^14^ Sharipova *et al.* investigated the initial stages of SSD of Au thin films on a substrate with natural roughness (stemming from the grain structure of the oxidized surface). Although hole formation is faster compared to flat substrates, a highly porous yet continuous Au film was stabilized at later stages when the film thickness approached the average distance between the sharp edges of the cuboid oxides on the substrate (∼50 nm). For lower film thicknesses (∼5 nm) the film further dewets, leading to the formation of particles.^15^ Recently we reported that Pt particles formed by SSD on topographic, polycrystalline FTO substrates is a highly promising system for electrocatalysis. We shed light particularly on the role of the particle size distribution on the electrocatalytic activity,^9^ while the mechanism of SSD on FTO is thoroughly addressed in the present study.

A powerful tool to get insights into the mechanisms of SSD is *in situ* electron microscopy, as demonstrated by Niekiel *et al.*^16,17^ These studies facilitated the quantification of SSD kinetic parameters and the development of a model by revealing that the retraction of finger-like structures is the dominant morphological mechanism. Additionally, they provided insights into the interplay between grain growth, texture evolution, and the SSD process. Therefore, in the present study, we utilize *in situ* electron microscopy of plan-view samples, in combination with rapid thermal annealing protocols and *ex situ* SEM, and AFM characterization, to shed light on the mechanism including early stages of SSD of Pt thin films on topographic FTO substrates. We observe that hole formation in the Pt films and consequently the initiation of Pt SSD starts in the FTO valleys, while competing Pt grain growth takes place on the terraces of the substrate. These mechanisms lead to a bimodal size distribution with small Pt particles located in the valleys and larger ones on the terraces. The study lays the ground to utilize the topography of practical substrates for tailoring the particle size distribution of *e.g.* nanoparticle electrodes.

## 2. Experimental section

### 2.1 Sample preparation

The studied Pt films (nominal thickness ∼5 nm) were magnetron sputter coated (ATC Poaris, AJA International Inc., USA) on FTO-coated glass substrates (TEC T10, Visiontek System, UK, surface roughness 20 nm) using a plasma power of 5 W and at a working pressure of 4.2 × 10^−6^ bar. Prior to Pt sputtering, the FTO-coated glass substrates were ultra-sonically cleaned for 15 min in acetone followed by isopropanol, and ethanol, respectively (all solvents technical grade, BOOM BV). After the cleaning, substrates were finally rinsed with Milli-Q water (18.2 MΩ cm) and dried.

For *ex situ* electron microscopy investigations of the early stages of SSD, the Pt films on FTO were thermally annealed in Ar/H_2_ (5%) atmosphere with a heating ramp of 100 °C s^−1^ using a rapid thermal annealing (RTA) furnace (AS-One 100, Annealsys, France) at 500 °C for different durations.

A cross-sectional lamella of the as-deposited state was prepared using the focused ion beam (FIB) lift out method (Helios Nanolab Dual FIB/SEM, Thermo Fisher USA). To achieve electron transparency, the samples were thinned with a Ga+ beam until thickness of ∼90 nm by lowering the acceleration voltage subsequently from 30 kV down to 1 kV.

Additionally, TEM samples in plan-view were prepared. Therefore, discs with a diameter of 3 mm were cut out of the Pt-FTO heterostructures (as-deposited and 20s RTA annealed) with an ultrasonic cutter (Model 601, Gatan), which were then ground to a thickness of 100–120 μm using a manual grinding machine (EcoMet 30, Buehler) starting with P1200 silicon carbide paper (Struers), followed by 9 μm diamond lapping foil (Allied). The center of the disc was then further thinned down to 15–20 μm with a dimple grinder (Model 656, Gatan). As a final step, ion milling was carried out at an angle of +6°/−6°. First milling with 3 kV for 6.5 h was utilized which was finished polishing with 1 kV for 1.5 h using a Precision Ion Polishing System (PIPS II, Gatan) until a small hole could be detected in the center of the sample-disc.

### 2.2 Characterization

For *in situ* (scanning) transmission electron microscopy ((S)TEM) investigations, a Titan Themis^3^ 300 microscope (Thermo Fisher, USA) operating at 300 kV and equipped with a Gatan furnace heating holder system model 652 was utilized. To study the structural evolution of the Pt thin-films, the prepared plan-view samples were used. During the *in situ* experiment, the samples were stepwise heated up to 700 °C. *Ex situ* STEM imaging and energy dispersive X-ray spectroscopy (EDS) measurements were conducted with a Thermo Fisher Spectra 200 C-FEG (Thermo Fisher, USA) operated at 200 kV with an convergence angle of 20 mrad. For the *ex situ* SEM studies, a Helios Nanolab Dual FIB/SEM (Thermo Fisher, USA) was utilized using backscattered electrons for imaging.

The particle sizes of the 1 h and 2 4 h samples were determined by segmenting four STEM images per sample using ImageJ software, which provided the particle areas in nanometers (nm^2^). The equivalent circle diameter (ECD) was then calculated from these areas and used as the particle diameter. Additionally the Feret's diameter (*D*_f_) was calculated from the segmented data and compared to the ECD.

Atomic force microscopy (AFM) measurement was carried out in non-contact mode using a JPK Nanowizard 4 Nanoscience microscope for Pt-FTO. The cantilever was Tap 150-G from BudgetSensors with a resonance frequency of 150 kHz. The images were acquired with a line rate of 0.5 Hz. For determining the roughness of the as-deposited Pt-films on SiO_2_, the topographical analysis was performed using atomic force microscopy (AFM) on a Bruker Dimension Icon system operating in Tapping Mode. BudgetSensors TAP300-Al probes were employed for high-resolution surface imaging.

Additionally, X-ray reflectivity (XRR) measurements were carried out with a X'Pert reflectometer for determining the roughness of as-deposited Pt films on SiO_2_ substrates. The XRR curves were fitted with a 2-slab model (Si and Pt) utilizing GenX.

## 3. Results and discussion

**Fig. 1:**
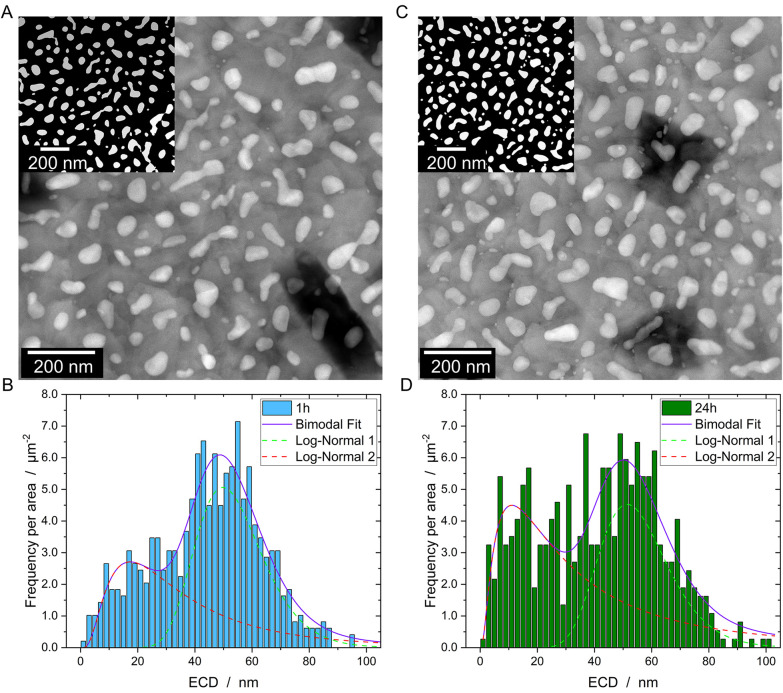
HAADF-STEM as well as segmented images (inset) of Pt particles after annealing at 500 °C for 1 h (A) and 24 h (C). In (B) and (D), the particle size distribution of samples heated for 1 h and 24 h are shown, revealing a bimodal distribution for both samples. A comparison of ECD with the Feret's diameter is shown in the ESI (Fig. S1†).

Conventionally prepared plan-view samples were examined using high-angle annular dark field (HAADF) STEM imaging to obtain a detailed view of the particle size distribution, including particles located deep within the FTO valleys, as previously observed by SEM imaging.^9^ For quantitative analysis, the images were recorded and subsequently segmented, as illustrated in Fig. 1A (1 h annealing at 500 °C) and Fig. 1C (24 h annealing at 500 °C). The analysis of the particle distribution, as depicted in Fig. 1B and D confirms a bimodal particle distribution for both annealing times where the small particles are located in the valleys of the FTO grains and the large particles on the flat FTO facets. For analyzing the particle size for each fraction, the particle size with highest frequency *x*_c_ was determined using a bimodal log-normal fit (*cf.*Table 1). Notably, for the longer annealing time (24 h), a shrinkage of the smaller particle fraction is observed as well as a small increase in the fraction of small particles, whereas the size of the larger particles slightly increases. While the latter is a common fingerprint of Ostwald ripening,^18^ the former suggests that still morphological changes due to SSD are ongoing like separation of bicrystalline particles into single particles. It should be noted that some particles exhibit irregular shapes. To assess whether the ECD remains a reliable approximation of particle size, the Feret's diameter (*D*_f_) was also extracted from the segmented data (see Fig. S1 and Table S1†). Although showing small deviations in the absolute values, the conclusion, which is shrinkage for the small and growth of the large particle fraction, drawn from the analysis of the ECD above are confirmed.

**Table 1:** Fit parameter from the particle size distribution, where *x*_c_ presents the mode of the log normal fit (particle size with highest frequency)

	*x* ^small^_c_/nm	*x* ^large^_c_/nm	Fraction small NPs/%
1 h	17.4 ± 5.4	49.8 ± 0.9	29.8
24 h	11.1 ± 5.7	51.4 ± 1.7	38.5

**Fig. 2:**
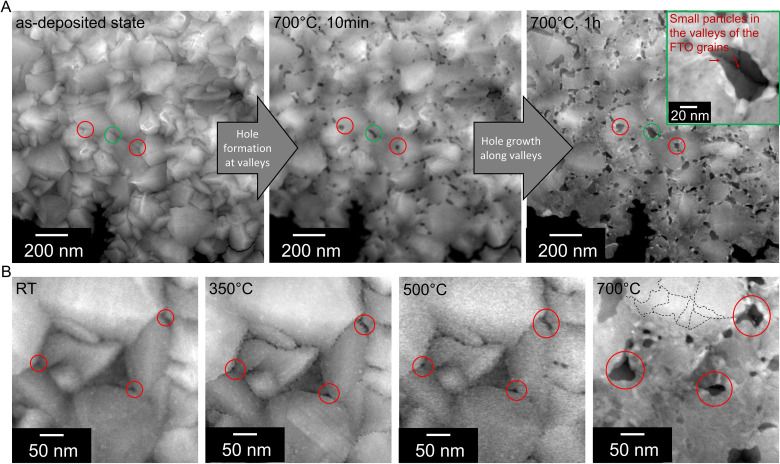
STEM *in situ* heating experiment. (A) Overview images depict the evolution of the Pt film from the as-deposited state (left) to heating at 700 °C for 10 minutes (middle) and 1 hour (right). The image series clearly reveals that hole formation is initiated in the valleys of the FTO and further expands along the valleys. (B) Higher magnification images reveal that, even before annealing pre-existing holes at grain boundary triple junctions in the valleys of the FTO are present, these holes grow larger as the temperature increases.

To get insight into the SSD mechanism, as-deposited thin films were conventionally prepared in plan-view geometry and investigated *via in situ* STEM (*cf.*Fig. 2). In (A) a time series tracking the Pt-film evolution at 700 °C is shown. After 10 min, holes (areas of dark contrast where the FTO substrate is exposed) are clearly visible at the valleys of the FTO substrate. During further annealing for up to 1 h, the holes grow along the valleys of the substrate, leaving behind small particles preferentially located at the grain boundaries of the FTO substrate (see green box). In (B), higher magnification images provide further insights, showing that the initiation of SSD within the valleys is influenced by pre-existing holes in the as-deposited state (see red marks), likely caused by shadowing effects during the Pt film deposition by magnetron sputtering. Tracking the temperature series from 350 °C to 500 °C and finally 700 °C reveals that SSD primarily progresses at these pre-existing holes, as they continue to grow, and edge retraction is concentrated in these regions, highlighted with red circles.

**Fig. 3:**
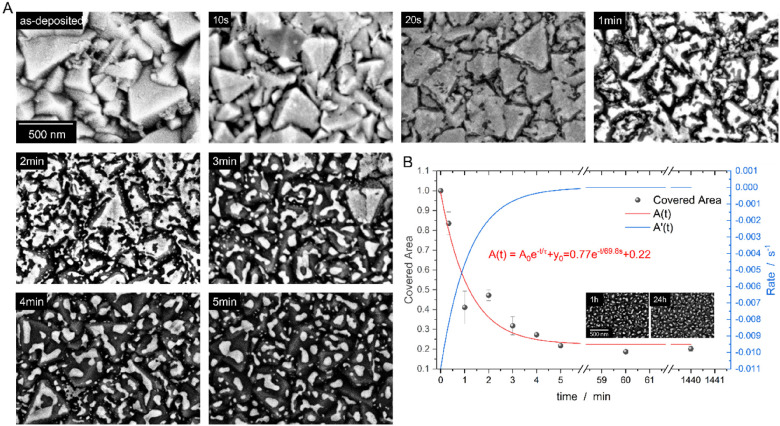
SEM images of the RTA heating series (A) and the corresponding analyzed area, covered with Pt (B), complement the TEM data (Fig. 1) and clearly illustrate the formation and growth of holes at the valleys of the FTO. After 5 minutes, the SSD process is already in its final stages, as evidenced by comparison with samples heated for 1 h and 24 h (from Harsha *et al.*^9^).

To avoid the impact of vacuum as well as the relatively slow heating rates necessary to overcome the thermal drift of the utilized conventional TEM heating holder during *in situ* investigations, RTA experiments were conducted at 500 °C in Ar atmosphere (Fig. 3). In this context, the RTA capabilities allow to investigate heating times down to a few seconds^19,20^ shedding light on the very first stages of SSD, while keeping the same conditions (temperature and atmosphere) as in our previous studies.^9^ The SEM data (Fig. 3A) is in line with the *in situ* TEM data, confirming the onset of SSD in the valleys. Additionally, two timescales are observed: (i) in the valleys, SSD kinetics is fast, with the film retracting from the valleys (visible as dark contrast around the large FTO grains) shortly after 20 s, and (ii) on the FTO terraces, SSD kinetics is slow, eventually leading to the bimodal particle distribution. The SEM images in Fig. 3A, along with the covered area plot in Fig. 3B, demonstrate that the SSD process in the valleys and on the terraces is nearly complete after just 5 min. This is further supported by Fig. 3B, showing an exponential decay of the covered substrate area. The trend also aligns well with the long-term behavior (1 and 24 h) observed with samples heated in a conventional furnace. In this context, almost no difference in covered substrate area can be detected when comparing the 5 min sample with the long annealing times. This is not surprising, since the rate of substrate exposure drops to ∼1 × 10^−4^ s^−1^ already after 5 min (Fig. 3B). The plot of the covered area and the rate of substrate exposure, together with the Pt-morphology observable after 5 min (Fig. 3), as well as 1 h and 24 h (Fig. 1) demonstrates that the film is largely dewetted (as indicated by the low covered area of ∼20%) but has not yet completely equilibrated into particles—evidenced by the presence of individual particles alongside elongated conjoint particle structures. The fact, that the NPs are of irregular shape and do not reach its equilibrium crystal shape (ECS) can be attributed to (i) the topography of the substrate and (ii) the ratio between processing temperature, *T*_p_ and the melting temperature, *T*_m_ of Pt with *T*_p_/*T*_m_ = 0.28 is relatively low. The relatively low *T*_p_ of 500 °C was chosen to ensure the integrity of the FTO support (limit at ∼550 °C (ref. 21)) also for long heating times. A comparison of the FTO before and after annealing is shown in Fig. S2A†*via* cross-sectional FIB lamellae. The STEM images reveal, that no fracture or morphological changes occur during annealing, even after 24 h. Additionally, a STEM EDS measurement is presented (Fig. S2B†) documenting the absence of fluorine in the NPs, further confirming the integrity of the FTO substrate.

Worth noting, the SSD kinetics in the *in situ* experiment were much slower particularly at the terraces of the FTO grains. Two reasons are identified for the different behavior. First, the different atmospheres comparing TEM with RTA. In TEM, the vacuum level is typically ranging from 5 × 10^−7^ mbar to 5 × 10^−8^ mbar, whereas in the RTA the chamber is first evacuated to 1 × 10^−4^ mbar before a purging to atmospheric pressure (atm) which takes place for better heat conductance during annealing. This implies that in the RTA, more residual oxygen is present due to (i) the lower vacuum environment and (ii) additional residual oxygen might be introduced during purging to atm. The higher level of residual oxygen might lead to an enhanced atom mobility, as described in the literature.^22–24^ Additionally, in the RTA, a mixture of Ar/H_2_ is used during annealing, which further can enhance surface self-diffusion of Pt atoms by adsorbed hydrogen.^25^

Second, besides the different atmosphere (vacuum in TEM and Ar/H_2_ in RTA), this effect mostly stems from the competition between grain growth and SSD.^1,17^ As depicted in Fig. 2B large grains are present (marked with dashed lines) at 700 °C. The slow heating rates in the *in situ* experiments lead to grain growth already at low temperatures before SSD takes place, thus SSD is mostly suppressed on the terraces of the FTO support.

**Fig. 4:**
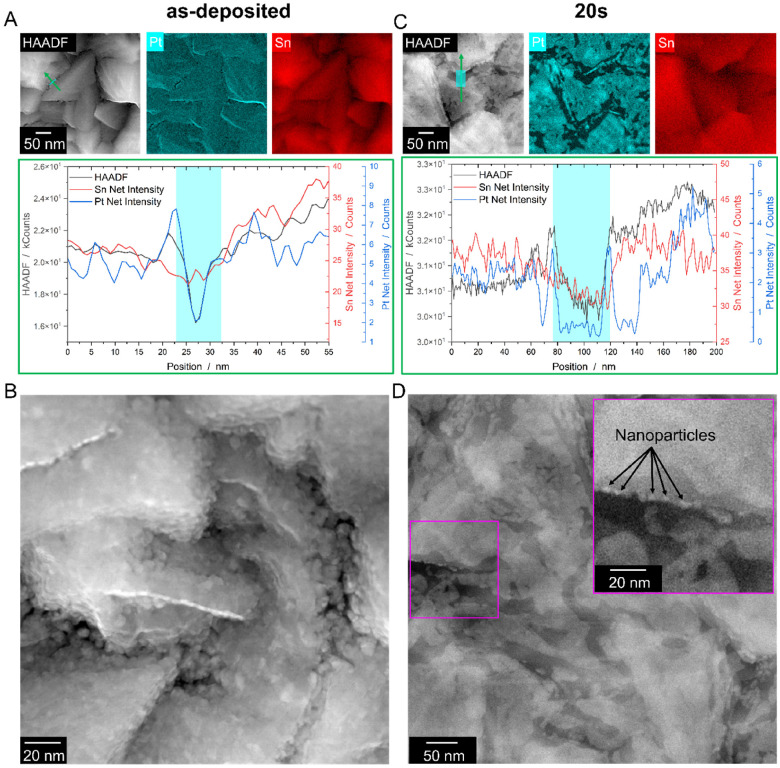
STEM HAADF image and EDS maps (top) and a line profile across a FTO valley (bottom) on a plan-view lamella of (A) the as-deposited sample showing pre-existing holes, indicated by the drop in Pt and HAADF signal (highlighted cyan area) and documented by a higher magnification HAADF image in (B). In (C) the EDS maps of a sample after annealing for 20 s in the RTA is shown. The line profile reveals that Pt is retracted from the inclined facet, while at the top edge and within the valley Pt rims are present (increased Pt signal). The magnified view in (D) confirms the retraction at inclined facets and reveals that in the valleys, already small nanoparticles have formed.

To investigate the mechanism, conventionally prepared plan-view TEM samples of an as-deposited Pt/FTO sample and a dewetted sample after heat treatment in RTA for 20 s were prepared (*cf.*Fig. 4A and B). The HAADF images, which are sensitive to mass-thickness contrast,^26^ reveal a dark contrast in the FTO valleys, which indicates that, at these locations, less Pt interacts are present, *i.e.*, either the Pt film is thinner, or the FTO substrate is even exposed. Additionally, EDS mapping reveals a reduced Pt signal in the FTO valleys, which is further confirmed through an EDS line profile (Fig. 4A bottom). The drop in the HAADF intensity profile can be attributed to a decrease in both the Pt and Sn signals. While the decrease in the Pt signal suggests a thinner Pt film on the inclined facets, the lower Sn signal indicates a change in the thickness of the FTO grain. The peak in the Sn signal deep in the valley corresponds to the exposed FTO substrate, likely caused by a pre-existing hole in the Pt film. This is also evident from the higher magnification image shown in Fig. 4B, which reveals a microstructure dominated by small grains (∼5 nm) and a non-closed thin film with pre-existing holes at the valleys of the FTO grains. To evaluate the roughness of the as-deposited film, Pt films were deposited on flat SiO_2_/Si wafer with the same deposition parameter described in section 2.1. AFM as well as XRR measurements revealed a very low roughness of ∼0.35 nm (*cf.* Fig. S3†). The reason for the pre-existing holes might be the underlying growth mechanism of thin films produced *via* PVD. The growth mode is likely island growth, generally described as Volmer-Weber growth.^27^ Due to a reduced film thickness within the valleys (*cf.*Fig. 4B), no complete coalescence of the film can happen, leading to pre-existing holes. Fig. 4C shows the EDS data of a plan-view Pt-FTO sample prepared after annealing for 20 s. The data demonstrates the retraction of Pt from inclined facets, leaving behind a rim at the edge of the grain (left side of the cyan box, Fig. 4C bottom) and a rim in the valley of the FTO landscape (right side of the cyan box, Fig. 4C bottom), both indicated by a peak in the Pt-EDS signal. Between the rims, the Pt signal nearly drops to zero, indicating a complete retraction of Pt from the FTO facet. Meanwhile, the scattering (HAADF) signal follows the chemical FTO signal (Sn line-profile). A closer look at the structures formed in the valleys discloses that small nanoparticles have already formed after the short annealing time (Fig. 4D), similar to the *in situ* experiments.

**Fig. 5:**
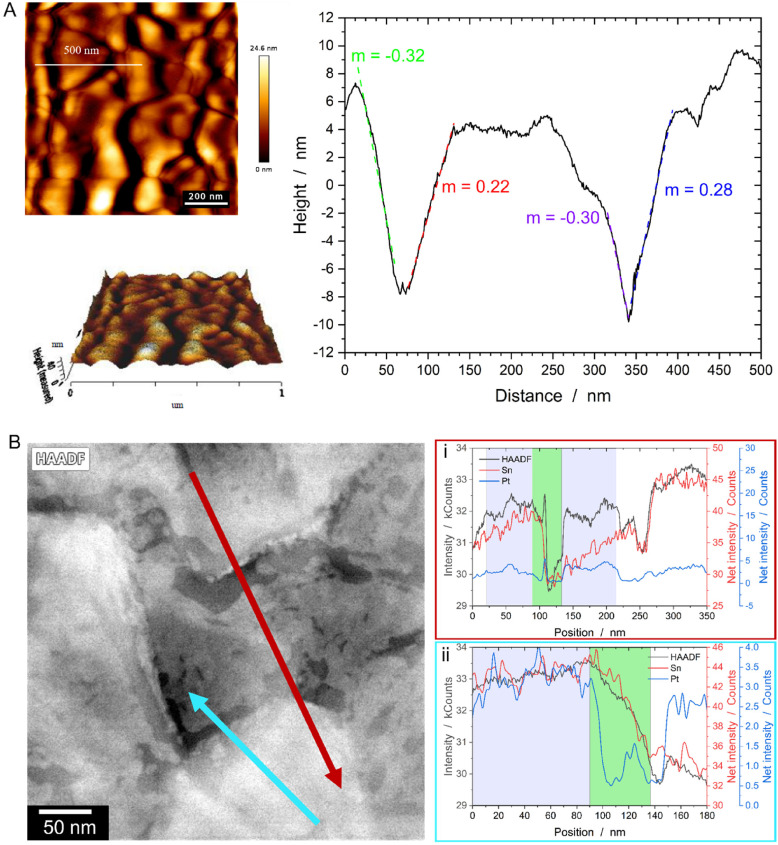
(A) AFM measurements revealing the morphology of the FTO consisting of steep terraces with slopes of about 30% as well as flat terraces. (B) STEM EDS mapping of the 20 s RTA-annealed sample confirming the FTO morphology measured by AFM *via* extracted linescans. In both line profiles, the sharp drop in the HAADF signal corresponds to a significant decrease in the Sn signal, signifying a rapid thickness change due to the steep nature of the Sn terraces (green box), while the purple-marked areas represent plateau-like terraces. Furthermore, a reduction in the Pt signal is observed on the inclined terraces, whereas peaks in the net intensity signal arise from Pt rims directly located in the valleys.

For characterization of the intrinsic FTO topography, AFM measurements of the bare substrate have been conducted, revealing grains with both horizontal and inclined facets (Fig. 5A). Similar information is obtained from the STEM EDS line scans displayed in Fig. 5B. Both line profiles indicate that sharp drops in the HAADF signal correspond to a significant decrease in the Sn signal (green areas), while the purple-marked areas represent plateau-like terraces characterized by a constant HAADF as well as Sn signal. A correlation with the Pt signal shows that Pt already retracted from the steeply inclined facets, while the plateaus remain covered, as evidenced by a constant Pt signal. Accelerated SSD on the steep facets is likely due to two reasons: (i) a thinner initial film thickness resulting from geometric or shadowing effects during magnetron sputter deposition^28^ and (ii) surface curvatures inherent to the topographic FTO substrate. For the former, AFM line scans (Fig. 5A) as well imaging of a cross sectional FIB lamella (Fig. S4†) reveal inclination angles of the terraces ranging from ∼15° to nearly 90°. At an inclination angle of about 15°, the expected film thickness should be around 97% of that on a flat substrate, as predicted by geometric considerations (the cosine law of film-thickness reduction, see ESI and Fig. S5† for details). However, the valleys’ relatively high aspect ratio of 0.7 (see ESI†) restricts the line-of-sight paths, leading to a reduced Pt flux during sputtering and, consequently, significantly thinner Pt films at the valleys base.^28,29^ For the surface curvatures mentioned in (ii), we propose, in line with literature, that an additional driving force facilitates Pt diffusion from peaks and ridges (positive local curvature) to valleys (negative local curvature).^14,30,31^ This behavior is known as Gibbs-Thomson effect, which is fundamentally rooted in the theory of SSD. Essentially, the local excess chemical potential Δ*μ* varies with the local curvature *κ* of the film, following the relation Δ*μ* = *κγΩ*, where *γ* represents the surface energy and *Ω* the atomic volume. Consequently, this theory explains the preferential diffusion from curved to flat surfaces, driven by differences in excess chemical potential – a well-known concept from grain boundary grooving theory^32^ and SSD on pre-patterned substrates.^30,31^

**Fig. 6:**
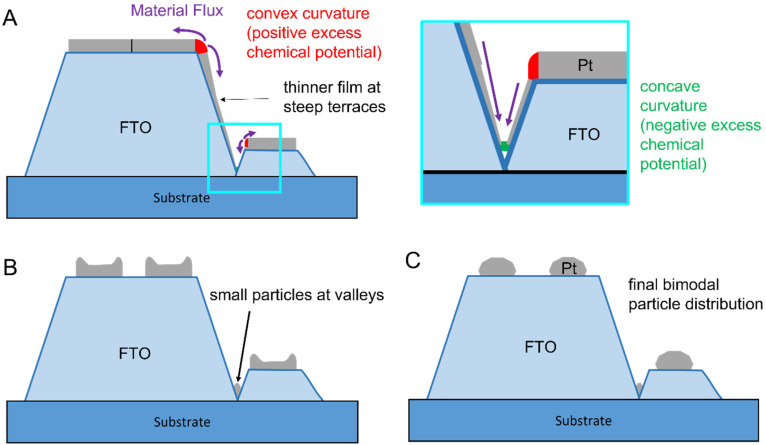
Schematic model of the SSD process for Pt on FTO: the top section (A) illustrates the microstructure of the as-deposited Pt film, influenced by the FTO topography. Convex curvatures at the grain edges (marked red) and concave curvatures in the valleys (marked green) are introduced by the topography. Shadowing effects further result in a slightly thinner Pt film on the inclined terraces compared to flat terraces. The surface curvatures drive material transport (Gibbs-Thomson Effect), where Pt diffuses towards the plateaus and valleys, causing island formation on the plateaus and the formation of small particles in the valleys (B). In the final stage (C), the islands on the plateaus merge into larger particles, resulting in a bimodal particle size distribution.

Considering both effects, we propose a model for SSD of Pt on topographic FTO, as illustrated in Fig. 6: at the edges of the FTO grains, the film exhibits a positive excess chemical potential due to its convex curvature (marked in red), while in the valleys, a negative excess chemical potential arises from the concave curvature (marked in green). To minimize local energy, Pt flows from the edges to the center of the flat facets of the FTO grains and to the valleys. This process results in island formation on the plateaus and in the valleys. The particles in the valley (i) form earlier due to a thinner initial film thickness or pre-existing holes as initiation sites for SSD, and (ii) remain significantly smaller due to the lower Pt availability in the valleys compared to flat surfaces of the upper terraces (Fig. 6B). With continued annealing, the islands on the plateaus coalesce into larger particles, leading to the final bimodal particle distribution (Fig. 6C).

## 4. Conclusions

Overall, we have presented a methodology that combines plan-view sample preparation with *in situ* TEM analysis, providing a detailed understanding of the mechanisms and resulting morphologies of dewetted Pt particles on polycrystalline topographic FTO – a substrate relevant for applications such as electrochemistry. Notably, this methodology is widely applicable to virtually any dewetted system of interest, offering insights into mechanisms that remain inaccessible through conventional approaches used to study SSD.

Here, RTA, combined with plan-view sample preparation for *in situ* TEM analysis, provides unique insights into the initial stages of the SSD mechanism, even enabling the capture of dynamics occurring within the very first few seconds of the process. We demonstrate the formation of a bimodal Pt particle configuration, where small particles rapidly emerge in the valleys, while larger particles evolve over a longer timescale on the flat FTO terraces, competing with Pt grain growth. The Pt behavior in the valleys is driven by a reduced initial film thickness (≪5 nm) resulting from shadowing effects during film deposition. This leads to SSD within short time scales (seconds) in the valleys of the FTO support. In addition, the local surface curvature induced by the FTO grain topography drives Pt agglomeration both into the valleys and towards the center of the flat terraces, effectively separating the phenomenon into two distinct SSD processes. Due to the different Pt reservoirs, determined by the initial Pt thickness and the available area on the valley sides *versus* the flat terraces, the final particle size in the valleys is significantly smaller compared to that in the locally flat FTO regions. Thus, we demonstrate that topography can be strategically utilized to fabricate a fraction of small nanoparticles on a structured support. In Fig. S2A (top),† the microstructure of the FTO support is shown in cross-section, revealing its columnar growth, where grain size increases with film thickness. The pink line at 150 nm marks a threshold, suggesting that by stopping FTO film growth earlier, the structure and surface topography can be adjusted to achieve a finer grain structure with smaller terraces and steeper facets. This modification is expected to further enhance film breakup and yield an even higher density of smaller NPs. This increases the particle surface-to-volume ratio, potentially enabling stronger catalyst–support interactions and enhancing catalytic properties of nanoparticle produced *via* SSD.

## Author contributions

M. Dierner: writing – original draft, writing – review & editing, visualization, validation, investigation, formal analysis, data curation. J. Will: writing – original draft, writing – review & editing, supervision, data curation, conceptualization. S. Peters: resources. M. Wu: investigation. C. Rubach: resources. S. Harsha: writing – review & editing. R. K. Sharma: writing – review & editing. Z. Y. Siah: investigation. M. T. Abudukade: investigation. S. Ng: writing – review & editing. M. Altomare: writing – review & editing, funding acquisition, supervision, conceptualization. E. Spiecker: writing – review & editing, validation, supervision, resources, project administration, funding acquisition, formal analysis, conceptualization.

## Conflicts of interest

The authors declare that they have no known competing financial interests or personal relationships that could have appeared to influence the work reported in this paper.

## Supplementary Material

NR-017-D5NR00729A-s001

## Data Availability

The raw data pertaining to this study is available at Zenodo: 10.5281/zenodo.14860452.
