# Supplementary material for: Role of polycrystalline F–SnO2 substrate topography in formation mechanism and morphology of Pt nanoparticles by solid-state-dewetting
Source: Nanoscale. 2025 May 15;17(23):14338–47. doi: 10.1039/d5nr00729a (PMC12107527; doi:10.1039/d5nr00729a)
Supplement: NR-017-D5NR00729A-s001 [file NR-017-D5NR00729A-s001.pdf]

Supplementary Information (SI) for Nanoscale.
This journal is © The Royal Society of Chemistry 2025

Supporting Information

Role of Polycrystalline F-SnO_2 Substrate Topography on Formation Mechanism and Morphology of Pt Nanoparticles by Solid-State-Dewetting

M. Dierner[a], S. Peters[a], M. Wu[a], C. Rubach[a], S. Harsha[b], R. K. Sharma[b], Z. Y. Siah[c], M. T. Abudukade[b], S. Ng[c], E. Spiecker[a], M. Altomare[b] and J. Will[a*]

[a]Institute of Micro- and Nanostructure Research & Center for Nanoanalysis and Electron Microscopy (CENEM), Friedrich-Alexander-Universität Erlangen-Nürnberg, IZNF, Cauerstraße 3, 91058 Erlangen, Germany

[b]MESA+ Institute of Nanotechnology, University of Twente, Enschede 7500AE, The Netherlands

[c]Department of Chemistry and Pharmacy, Friedrich-Alexander-Universität Erlangen-Nürnberg, Egerlandstraße 3, 91058 Erlangen, Germany*Corresponding Authors

*Corresponding Author

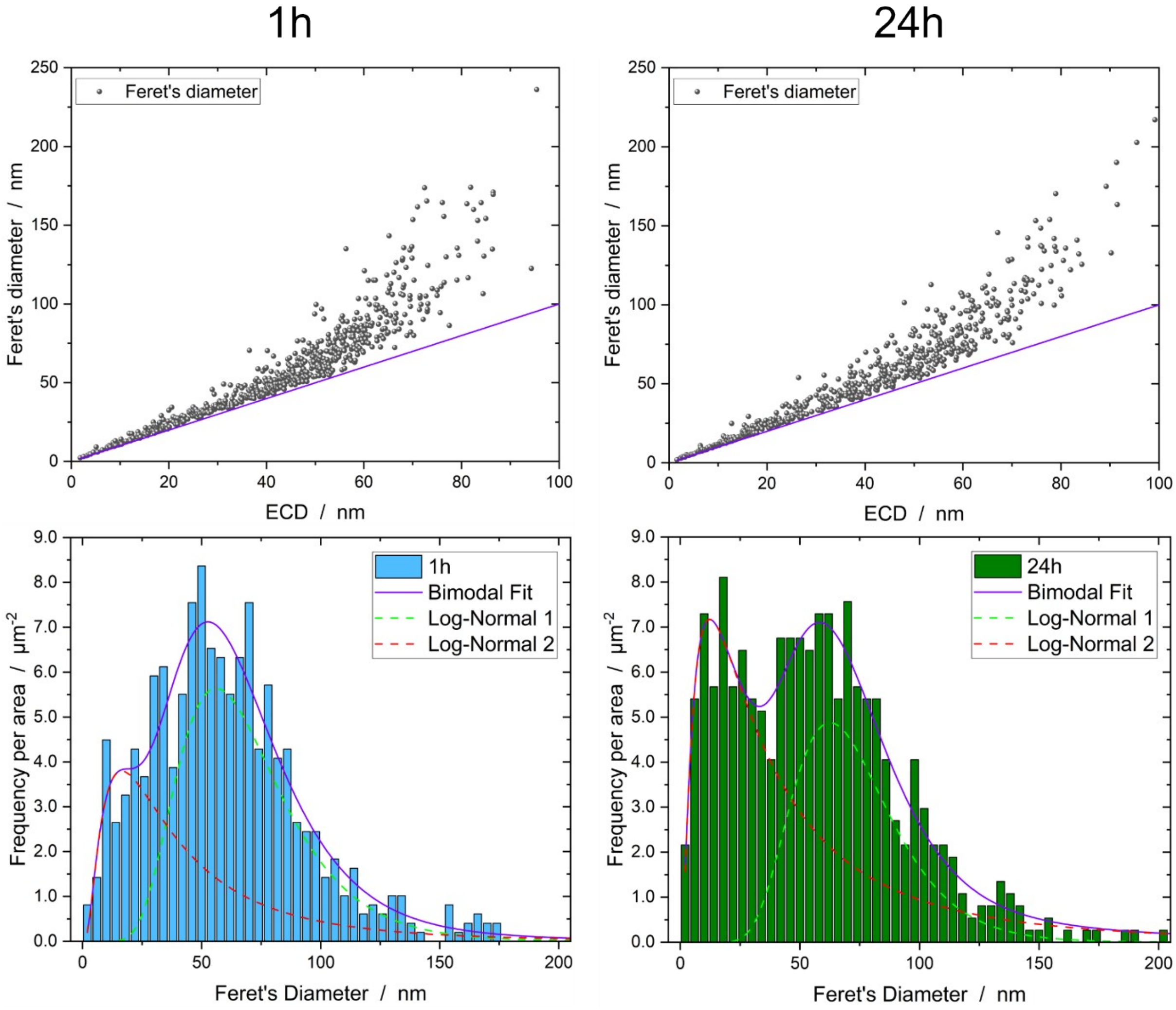

Fig. S 1: Top: Feret's diameter (D_f) versus the equivalent circle diameter (ECD). The deviation from a perfect circular particle is depicted by the deviation of the Feret's diameter (scattered data) from the straight line. This comparison demonstrates that the ECD is a suitable estimate of particle size, particularly for the smaller particles, as only minor deviations are observed Bottom: Particle size distribution using Feret's diameter.

Table S. 1: Fit parameter from the particle size distribution unsing Feret's diameter, where x_c presents the mode of the log normal fit (Particle size with highest frequency).

	x_c^{small} / nm	x_c^{large} / nm	Fraction small NPs / %
1h	16.5 ± 8.5	56.0 ± 2.5	24.6
24h	12.3 ± 2.9	62.6 ± 1.9	31.0

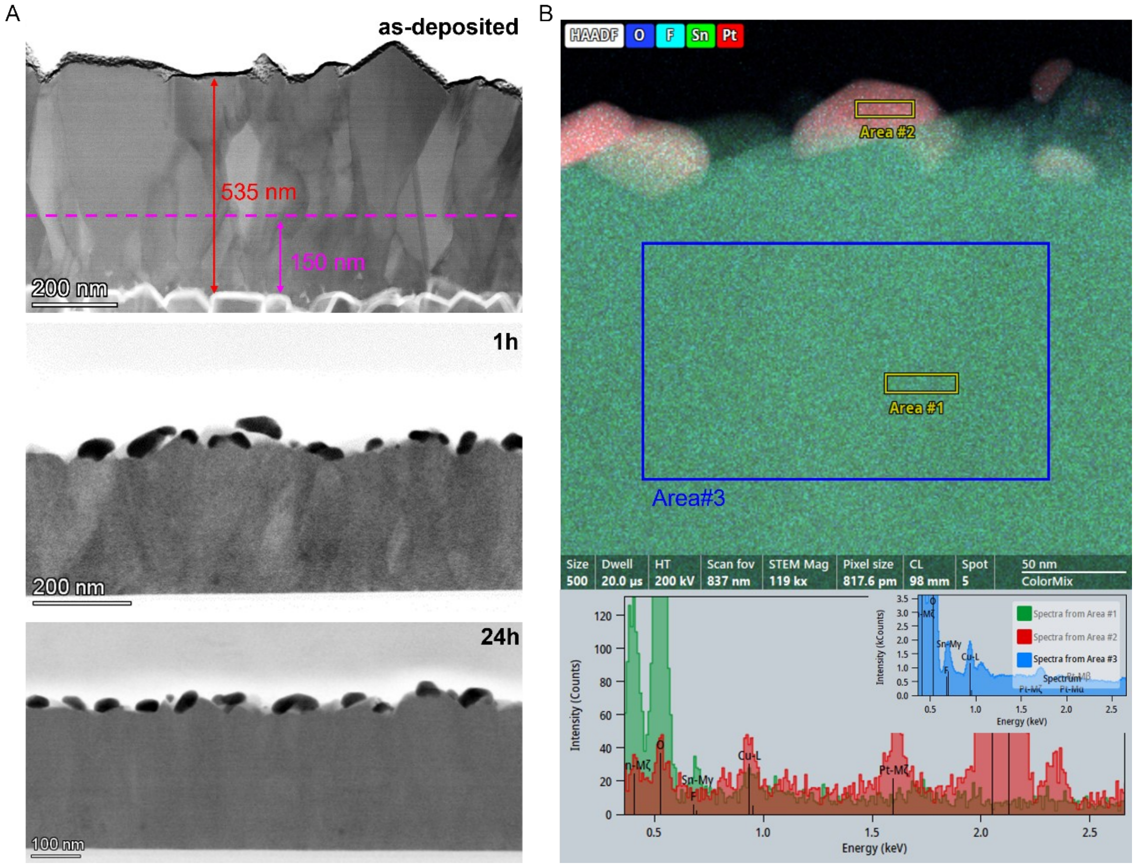

Fig. S 2: (A) Cross-sectional FIB lamellae of the FTO before (top) annealing and after annealing for 1h (middle) and 24h (bottom). The images clearly reveal that no fracture or morphological changes have occurred during annealing. (B) STEM EDXS of the Pt-FTO structure. While F is detected within the substrate, no F is present within the Pt NPs. For comparison, the same small area was used for generating the spectrum (bottom) than in the particle.

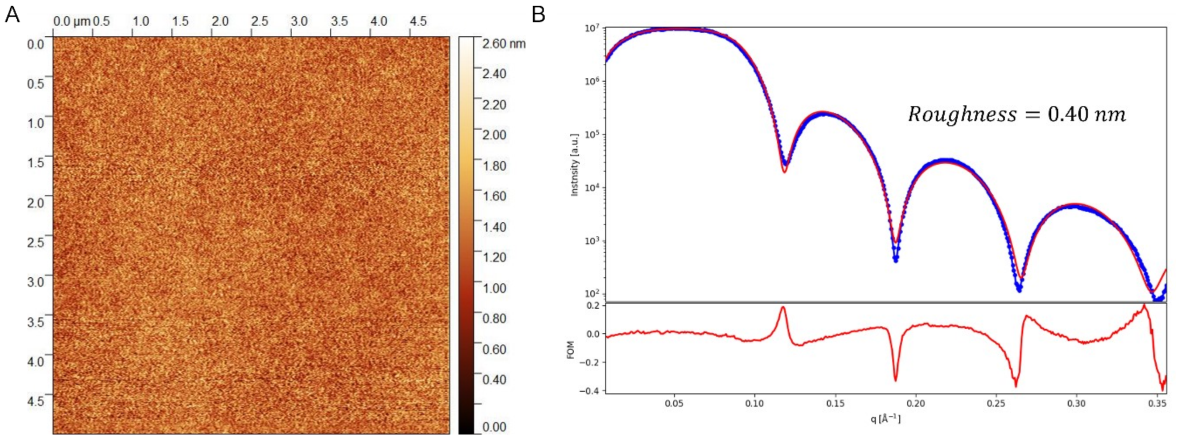

Fig. S 3: (A) AFM measurement of an ~5 nm thin as-deposited Pt film deposited on SiO_2-Wafer. The sputter deposition parameters were identical to the one used for the 5 nm Pt films deposited on FTO. The root-mean-square (RMS) roughness of the sample is determined to be 0.3 nm. (B) XRR measurement on a ~7.5 nm thin Pt film deposited on SiO_2. The roughness value is determined to 0.4 nm.

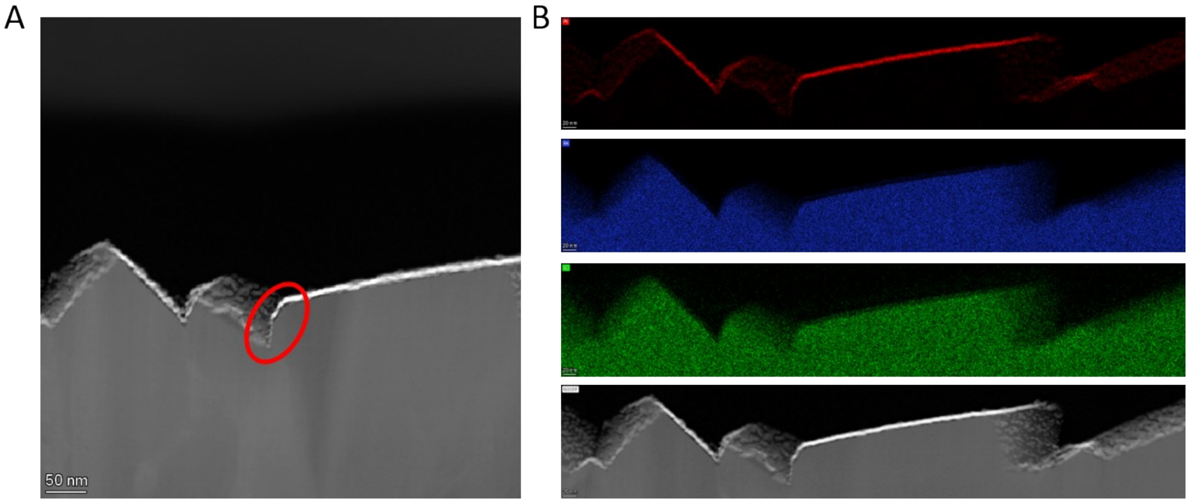

Fig. S 4: FIB cross-section of the as-deposited Pt-FTO sample, showing thinner Pt-films in steep regions

Approximation of shadowing effect during PVD

First, to consider the inclination of surfaces at magnetron sputtering, the cosine law of reduction can be used to approximate the shadowing effect related to the inclination angle:

$$t_{flat} = t_{inclined} \cdot \cos(\beta)$$

where t_{flat} is the film thickness at flat terraces, $t_{inclined}$ the film thickness at inclined surfaces and β the inclination angle.

In Fig.S2 the calculations are shown for inclination angles ranging from 0° to 90°.

Second, the aspect ratio (AR) of the valleys plays a significant role for shadowing effects, which is determined as follows:

$$AR = \frac{d_{Valley}}{w_{Valley}}$$

where d_{valley} is the depth and w_{valley} the width of the valleys.

If the AR is <<1 the shadowing effect would be small. With increasing AR, the shadowing effect gets stronger [22], [23].

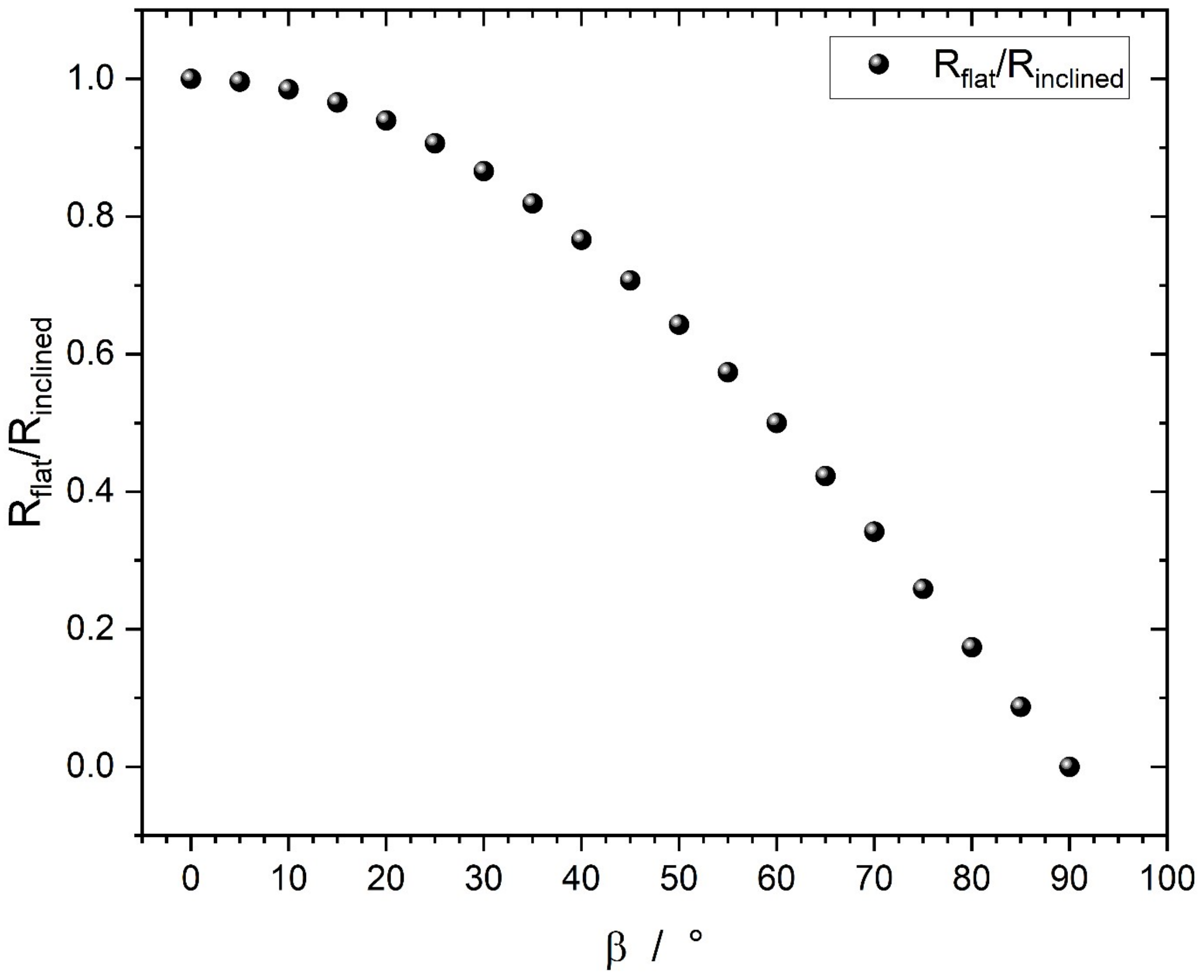

Fig. S 5: Cosine law of reduction effect on the film thickness of sputtered films
